# Dataset on quality and physiological changes of raspberry fruit during their development and under auxin *in-vitro* assay

**DOI:** 10.1016/j.dib.2018.10.089

**Published:** 2018-10-27

**Authors:** Liliam Monsalve, Aníbal Ayala-Raso, Maricarmen Bernales, Monika Valdenegro, Bruno Defilippi, Mauricio González-Agüero, Sam Cherian, Lida Fuentes

**Affiliations:** a Centro Regional de Estudios en Alimentos Saludables (CREAS), CONICYT-Regional GORE Valparaíso Proyecto R17A10001, Valparaíso, Chile; b Instituto de Estadística, Facultad de Ciencias, Universidad de Valparaíso, Gran Bretaña, 1093 Valparaíso, Chile; c Escuela de Agronomía, Facultad de Ciencias Agronómicas y de los Alimentos, Pontificia Universidad Católica de Valparaíso, Calle San Francisco s/n, Quillota, Chile; d Unidad de Postcosecha, INIA La Platina, Santiago, Chile; e Agrifarm consultant, PWRA 68, Kakkanad West PO, Kochi 30, Kerala, India

## Abstract

The data presented in this article are related to the research article entitled “Expression of two indole-3-acetic acid (IAA)-amido synthetase (GH3) genes during fruit development of raspberry (*Rubus idaeus* Heritage)” (Bernales et al., In press). This data article describes the relation of all size variables between them and with the weight showing an increasing trend between length and weight and an inverse relation of fruit firmness and ethylene production during development. In addition, IAA treatment during auxin *in-vitro* assay showed no significant changes in firmness, a significant increase of ethylene and respiratory production.

**Specifications table**

**Table:** 

Subject area	Agriculture, biology
More specific subject area	Fruit development
Type of data	Table and figures
How data was acquired	The fruit size and weight were measures using a caliper and analytical balance, respectively.
	The fruit firmness was measured using the Firm Tech II equipment (BioWorks Inc., Wamego, KS, USA) and data were expressed as Newton (N).
	Ethylene production was quantified in a gas chromatograph (Shimadzu 8A, Tokyo, Japan) equipped with a flame ionization detector, and the resulting data were expressed as µL ethylene kg^−1^ h^−1^.
	Respiratory production was determined using a CO_2_ detector (MAP Head space Gas Analyser, Bridge Analysers, USA), and the resulting data were expressed as mg CO_2_ kg^−1^ h^−1^.
Data format	Analysed data
Experimental factors	Data were obtained from different development stages and auxin *in-vitro* assay according to Bernales et al. In press [1].
Experimental features	The relation between quality and physiological parameter during raspberry development and significant differences between the treatment of *in-vitro* assay were determined using R Statistical Software [2]
Data source location	Raspberry (*Rubus idaeus* L.) Heritage fruits were collected from commercial orchards that are located in Chimbarongo (34°41′45.54S; 71°10′01.71W; 333 masl), Chile.
Data accessibility	*Data are with this article*
Related research article	Bernales, M., Monsalve L., Ayala-Raso A., Valdenegro M., Martínez J.P., Travisany D., Defilippi B., González-Agüero M., Cherian S., Fuentes L. Expression of two indole-3-acetic acid (IAA)-amido synthetase (GH3) genes during fruit development of raspberry (*Rubus idaeus* Heritage). Sci. Hort. “In press” [1]

**Value of the data**

• These data present information about quality and physiological changes during raspberry development and auxin *in-vitro* assay.
• Data are important considering the factor and timing that determine raspberry quality.
• These data can be used for estimating the timing and concentration of hormonal treatment for improved fruit quality characteristics.

## 1 Data

**Fig. 1:**
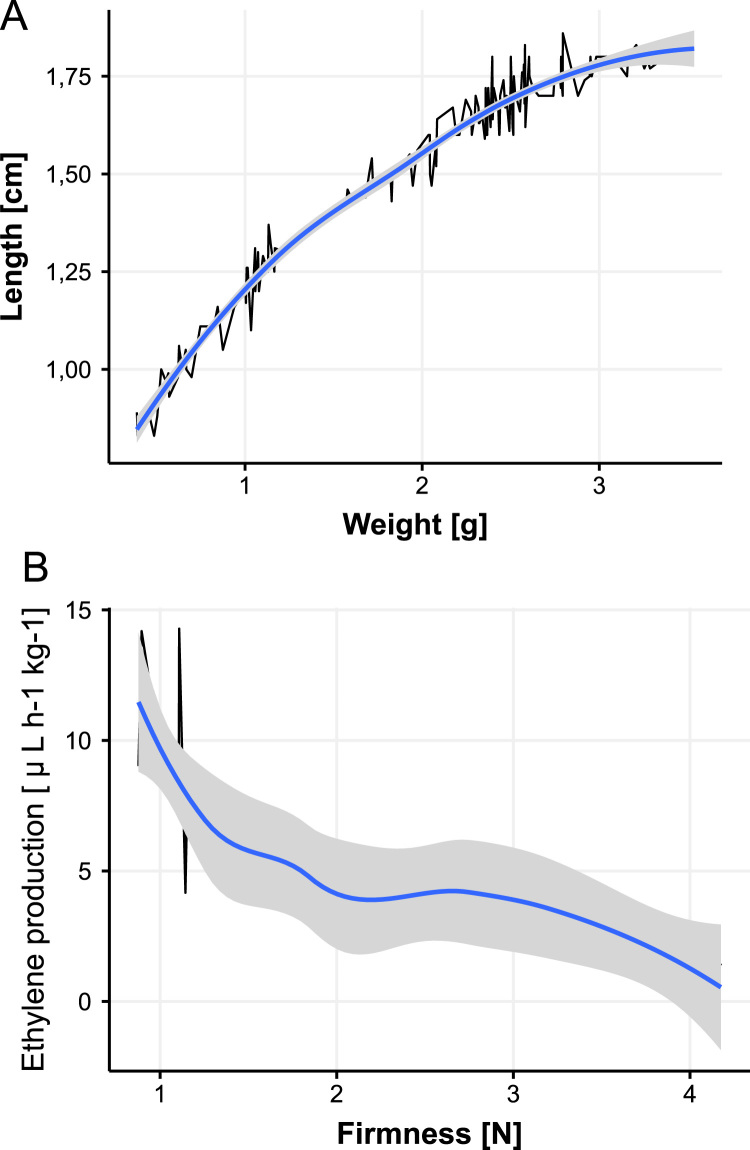
The relation between growth variables: length (cm) and weight (g) (A) and between fruit firmness (N) and ethylene production (µL kg^−1^ h^−1^) variables (B) for different developmental and ripening stages of raspberry were conducted. For growth variables, the relation matches the data presented in Table 1 showing a potential trend. The analysis included all samples (140 observations with four variables). On the other hand, the relation between fruit firmness and ethylene production matches a negative correlation, but with a polynomial fit curve. For both cases, for simplicity, a linear trend could use it. The analysis was considered for every ripening states of the raspberry fruit development.

A high correlation between all growth variables and weight was found (Table 1). The relation between length and weight variables shows a potential growth until 1.75 cm, and afterwards a less pronounced increment in fruit growth (after the fruits attained 2 g of weight) (Fig. 1A). On the other hand, the ethylene and fruit firmness showed a negative correlation (Table 1, Fig. 1B).

**Table 1:** Correlation between growth variables and between firmness and ethylene production variables for different developmental and ripening stages of raspberry.

**Correlation between growth variables**				
	**Superior diameter**	**Inferior diameter**	**Length**	**Weight**
**Superior diameter**	1.0000000	0.9439161	0.9720757	0.9704722
**Inferior diameter**	0.9439161	1.0000000	0.9404780	0.9332516
**Length**	0.9720757	0.9404780	1.0000000	0.9647104
**Weight**	0.9704722	0.9332516	0.9647104	1.0000000

**Correlation between ethylene and firmness variables**				
	**Firmness**	**Ethylene**		
**Firmness**	1.0000000	−0.7965057		
**Ethylene**	−0.7965057	1.0000000		

Analysis of growth variables included all samples (140 observations with four variables). Correlation between firmness and ethylene production considering every ripening stages of the raspberry fruit. All analyses were done by the Pearson method.

**Fig. 2:**
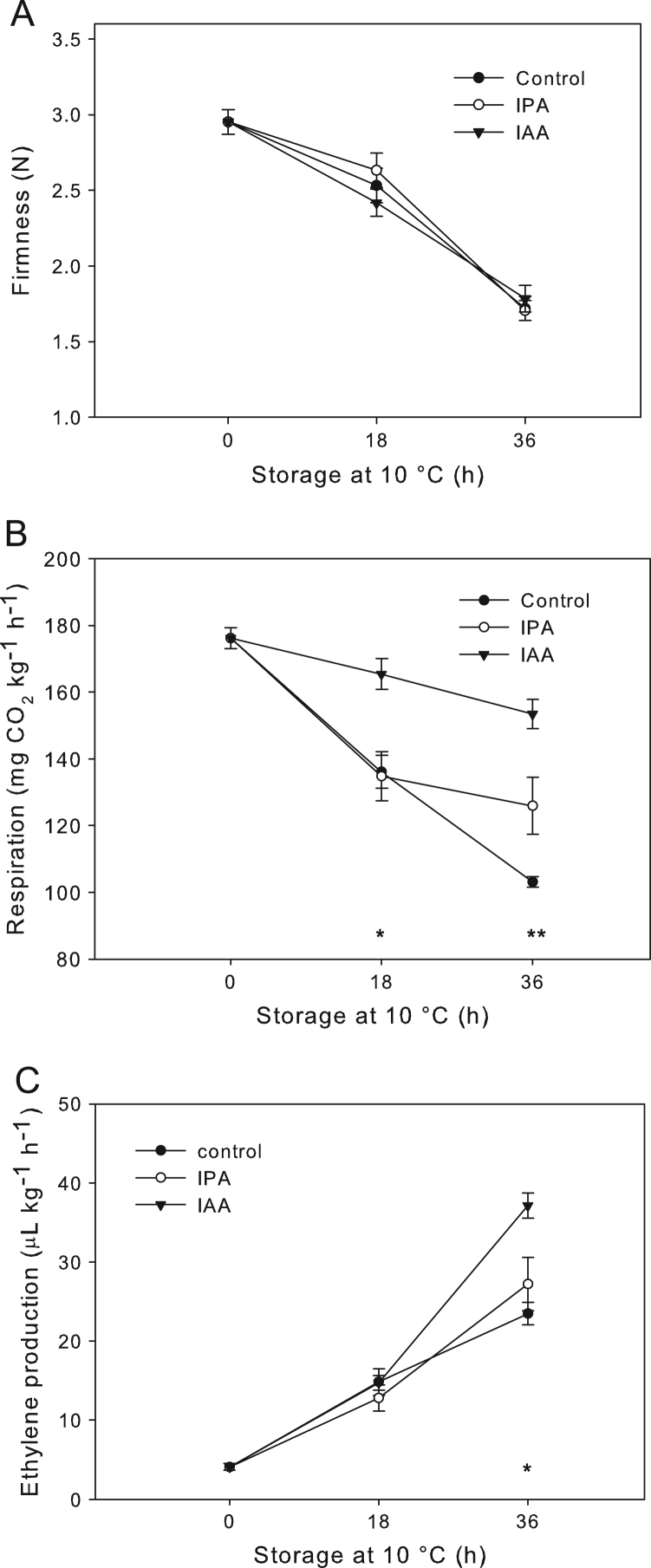
Physiological and quality parameters of raspberry fruit during auxin treatment. Auxin *in-vitro* assay was performed on W stage raspberry. (A) Firmness (N), (B) respiration mg CO_2_ kg^−1^ h^−1^ and (C) ethylene production (µL kg^−1^ h^−1^) were determined for IAA and controls samples. IAA: Indol-3-acetic acid; IPA: Indole-3-propionic acid. Data are represented as the means±SE from five replicates (each one contains 10 fruits). Significant differences from IAA- to control and IPA-treated fruits at the same time of treatment are indicated by (*) and (**) for the probability levels (*P* ≤ 0.05) and (*P* ≤ 0.01), respectively.

During auxin *in-vitro* assay, no significant differences of firmness were observed between the indole-3-acetic acid (IAA)-, indole-3-propionic acid (IPA)-treated and control fruit during the times evaluated (Fig. 2A). Conversely, respiration was significantly increased at 18 and 36 h by IAA treatment compared to IPA (no active auxin) and control conditions (Fig. 2B). Similarly, ethylene production was also increased at 36 h by IAA treatment (Fig. 2C).

## 2 Experimental design, materials, and methods

The quality and physiological assessment were determined during raspberry development and *in-vitro* auxin treatment [1]. Size variables and weight data for correlation analysis were obtained from twenty fruits of each developmental stage classified according to size and colour [1], [3], [4]. Data for auxin *in-vitro* assay were obtained analysing firmness, ethylene and respiratory production in three groups of treatments [1], [5].

The fruit size and weight during raspberry development were measures using a caliper and analytical balance, respectively. Fruit firmness, ethylene production and respiratory production during development and auxin *in-vitro* assay were determined as described by Bernales et al. [1], and the resulting data were expressed as the mean ± standard error (SE) of Newton (N), µL of ethylene kg^−1^ h^−1^ and mg CO_2_ kg^−1^ h^−1^, respectively. The data obtained during development were correlated by means of Pearson׳s correlation matrix. An analysis of variance was performed for data obtained from auxin in-vitro assay, and significant differences were determined at *P* ≤ 0.05 (*) and *P* ≤ 0.01 (**) (ANOVA test). All data were analysed using R Statistical Software [2].
