# Supplementary material for: Dataset on quality and physiological changes of raspberry fruit during their development and under auxin in-vitro assay
Source: Data Brief. 2018 Oct 27;21:1521–5. doi: 10.1016/j.dib.2018.10.089 (PMC6240639; doi:10.1016/j.dib.2018.10.089)
Supplement: Supplementary file 1 — Supplementary material. [file mmc1.pdf]

AUTHOR DECLARATIONFOR DIB-D-18-02630 MANUSCRIPT

Manuscript Title: Dataset on quality and physiological changes of raspberry fruit during their development and under auxin *in-vitro* assay

Authors: LiliamMonsalve[a], Aníbal Ayala-Raso[b], MaricarmenBernales[a], Monika Valdenegro[c], Bruno Defilippi[d], Mauricio González-Agüero[d], Sam Cherian[e], Lida Fuentes[a]

We wish to confirm that there are no known conflicts of interest associated with this publication and that the financial support for this work was CONICYT, whose objective is to finance research works and scientific publications.

We confirm that the manuscript has been read and approved by all named authors and that there are no other persons who satisfied the criteria for authorship but are not listed. We further confirm that the order of authors listed in the manuscript has been approved by all of us.

We confirm that there is not protection of intellectual property associated with this work and that there are no impediments to publication.

We understand that the Corresponding Author is the sole contact for the Editorial process (including Editorial Manager and direct communications with the office). He/she is responsible for communicating with the other authors about progress, submissions of revisions and final approval of proofs. We confirm that we have provided a current, correct email address which is accessible by the Corresponding Author and which has been configured to accept email from lfuentes@creas.cl

Signed by all authors as follows:

Name	Date	Signature
LiliamMonsalve	17-10-2018	Liliam Monsalve V.
Anibal Ayala-Raso	18-10-2018	[illegible]
MaricarmenBernales	17/10/2018	[illegible]
Monika Valdenegro	17/10/2018	[illegible]
Bruno Defilipi	October 17TH, 2018	[illegible]
Mauricio González Agüero	17-10-2018	[illegible]
Sam Cherian	17/10/2018	Sam Cherian
Lida Fuentes	18/10/2018	[illegible]

Scanned by CamScanner
